# *In vitro *proliferation of human osteogenic cells in presence of different commercial bone substitute materials combined with enamel matrix derivatives

**DOI:** 10.1186/1746-160X-5-23

**Published:** 2009-11-12

**Authors:** Christoph Reichert, Bilal Al-Nawas, Ralf Smeets, Adrian Kasaj, Werner Götz, Marcus O Klein

**Affiliations:** 1 Department of Orthodontics, Rheinische Friedrich-Wilhelms-University, Bonn, Germany; 2 Department of Oral and Maxillofacial Surgery, Johannes Gutenberg-University, Mainz, Germany; 3 Department of Oral and Maxillofacial Surgery, University Hospital Aachen, Aachen, Germany; 4 Department of Operative Dentistry and Periodontology, Johannes Gutenberg-University, Mainz, Germany

## Abstract

**Background:**

Cellular reactions to alloplastic bone substitute materials (BSM) are a subject of interest in basic research. In regenerative dentistry, these bone grafting materials are routinely combined with enamel matrix derivatives (EMD) in order to additionally enhance tissue regeneration.

**Materials and methods:**

The aim of this study was to evaluate the proliferative activity of human osteogenic cells after incubation over a period of seven days with commercial BSM of various origin and chemical composition. Special focus was placed on the potential additional benefit of EMD on cellular proliferation.

**Results:**

Except for PerioGlas^®^, osteogenic cell proliferation was significantly promoted by the investigated BSM. The application of EMD alone also resulted in significantly increased cellular proliferation. However, a combination of BSM and EMD resulted in only a moderate additional enhancement of osteogenic cell proliferation.

**Conclusion:**

The application of most BSM, as well as the exclusive application of EMD demonstrated a positive impact on the proliferation of human osteogenic cells *in vitro*. In order to increase the benefit from substrate combination (BSM + EMD), further studies on the interactions between BSM and EMD are needed.

## Background

The treatment of quantitative and qualitative defects of supporting bone tissue is one major aspect of modern dentoalveolar surgery and periodontology. In this context, alloplastic bone substitute materials (BSM) are well documented as alternatives to autogenous bone grafts for certain indications in the management of hard tissue deficiencies [1-5].

Various commercial BSM of different origin, chemical composition, and micro or macro-structural properties have been introduced and investigated in recent years [6-8]. Today, a large percentage of these BSM are based on calcium phosphate composites, such as hydroxyapatite (HA) and tricalcium phosphate (TCP), as well as bioactive glass (silicate: SiO_2_) [9]. In addition to the various well-employed substitutes with rather homogenous chemical compositions, such as Bio-Oss^® ^(HA), Cerasorb^® ^(β-TCP), and PerioGlas^® ^(SiO_2_), recent developments have focused on "composites" with different chemical phases, such as Straumann^® ^BoneCeramic (HA + β-TCP) [10], NanoBone^® ^(SiO_2 _+ HA), and BONIT^®^matrix (SiO_2 _+ HA + β-TCP) [11]. The latter biomaterials have been designed to combine the biological advantages of calcium phosphate and bioactive glass. Hence, the various BSM feature different biological behaviour *in vitro *and *in vivo *[12-14]. In a recent *in vitro *comparison of five commercial bone substitutes, Kuebler et al. [13] demonstrated significant differences among the investigated specimens with regard to osteogenic cell proliferation, pointing out the need for further research.

Emdogain^®^, a commercial mixture of porcine derived enamel matrix derivatives (EMD), is an evidence-based option for the treatment of bony defects in periodontal therapy [15-17]. Biologically active EMD ingredients are ligands, such as amelogenin, ameloblastin, enamelin, and tuftelin, that play a crucial role in the development of teeth and supporting structures [18,19]. A recent systematic review summarised the effect of EMD on relevant cell populations in the periodontal region, such as epithelial cells, gingival fibroblasts, periodontal ligament cells, osteogenic cells, and cementoblasts as stimulatory rather than inhibitory [18]. For osteogenic cells specifically, EMD have been shown to support cell viability and proliferation in a dose dependent manner [20,21], as well as encourage cell attachment [22], cell motility [23], and cell differentiation [22,24,25]. Recent studies of periodontal regeneration focused on the augmentation of BSM with EMD [5,26]. However, up to now, no significant clinical benefit could be measured, making further research on this approach desirable.

The application of either BSM or EMD into the hard tissue defect should ideally initiate and support tissue regeneration. For osteogenic cells, cell recruitment and migration into the defect (osteoconduction), and cell proliferation precede osteogenic cell differentiation [27], while cell proliferation plays a pivotal role for further successful regeneration. In cellular research, many biological assays focus on cell proliferation. The toxic or radioactive properties of assays like the H_3_-thymidin or BrDU assay are disadvantageous. The Alamar Blue^® ^assay is a well established, non-toxic, and non-radioactive method for continuously quantifying cellular proliferation over a long time interval [28].

The aim of this study was to compare the impact of various bone substitute materials on the proliferation of human osteogenic cells *in vitro*, employing the Alamar Blue^® ^assay over 7 days. Furthermore, the impact of the additional application of EMD on osteogenic cell proliferation activity was investigated.

## Materials and methods

### Cell Line

A commercial hip bone derived osteoblastic cell line (HHOBc, PromoCell, Heidelberg, Germany) was utilised. Cells were cultivated using a standard osteoblast cultivation medium, consisting of fetal calf serum (FCS, Gibco Invitrogen, Karlsruhe, Germany), Dulbecco's modified Eagle's medium (DMEM, Gibco Invitrogen), dexamethasone (100 nmol/l, Serva Bioproducts, Heidelberg, Germany), L-glutamine (Gibco Invitrogen), and streptomycin (100 mg/ml, Gibco Invitrogen). Cultivation was carried out at 37°C in a constant, humidified atmosphere with 95% room air and 5% CO_2_.

Prior to our experiments, the cell line was qualitatively characterised by the immunohistochemical expression of alkaline phosphatase (AP) and osteocalcin (labelled streptavidin-biotin/horseradish peroxidase). Cells were passaged at regular intervals, depending on their growth characteristics, using 0.25% trypsin (Seromed Biochrom KG, Berlin, Germany).

All trials were carried out at the 4^th ^cell passage. Osteogenic cells were detached and seeded on the different test substrates.

### Test Substrates and Incubation

Seven different commercial alloplastic BSM were investigated. Except for the biological sample derived from bovine bone (Bio-Oss^®^), all other samples were synthetic, composed of pure β-tricalcium phosphate (Cerasorb^® ^M, Bioresorb^® ^Macro Pore), pure bioactive glass (PerioGlas^®^), biphasic BSM (β-tricalcium phosphate + hydroxyapatite: Straumann^® ^BoneCeramic; silicon dioxide + hydroxyapatite: NanoBone^®^) or triphasic BSM (silicon dioxide + β-tricalcium phosphate + hydroxyapatite: Bonit^®^matrix). Table 1 provides a synopsis.

**Table 1:** Bone substitute materials investigated

Chemical composition and origin		**Abbr**.	C**ommercial name, manufacturer**	Investigated particle size, manufacturer's data
tricalcium phosphate: β-TCP	synthetic	CBM	Cerasorb^® ^M, Curasan	500-1000 μm
		
		BRE	Bioresorb^® ^Macro Pore, Oraltronics^®^	500-1000 μm

biological apatite: HA	bovine	BIO	Bio-Oss^®^, Geistlich	250-1000 μm

silicate: SiO_2_	synthetic	PGL	PerioGlas^®^, Sunstar Butler	90-710 μm

biphasic: β- TCP, HA	synthetic	BOC	Straumann^® ^BoneCeramic, Straumann	500-1000 μm

biphasic: SiO_2_, HA	synthetic	NBO	NanoBone^®^, Artoss	mean particle size: 600 μm

triphasic: SiO_2_, gβ-TCP, HA	synthetic	BIM	Bonit^® ^matrix, DOT	300 x 600 μm

(β-TCP: β-tricalcium phosphate, HA: hydroxyapatite, SiO_2_: silicon dioxide). Abbr.: Abbreviation. Manufacturers: Cursan AG (Kleinostheim, Germany), Oraltronics^® ^Dental Implant Technology GmbH (Bremen, Germany), Geistlich Biomaterials (Baden, Germany), John O. Butler GmbH (Kriftel, Germany), Straumann GmbH (Freiburg, Germany), Artoss GmbH (Rostock, Germany), DOT GmbH (Rostock, Germany).

The porcine derived protein mixture Emdogain^® ^(Straumann, Freiburg, Germany) was utilised as a commercial EMD.

In our investigation, 100 mg of the respective BSM were loosely placed into black 24 well plates (Thermo Fisher Scientific, Langenselbold, Germany), ensuring complete coverage of the well surface. Wells without BSM served as a control group. For those wells incubated additionally with EMD, an emulsion of 100 μg Emdogain^®^/ml was prepared and added to the respective wells. Osteogenic cells were added to the respective compositions at a density of 1*10^4 ^cells per well, and further cultivated at 37°C in a constant, humidified atmosphere of 95% room air and 5% CO_2_.

### Alamar Blue^® ^proliferation assay

The Alamar Blue^® ^(AB) assay (Biozol, Echingen, Germany) was performed according to manufacturer's guidelines for the quantification of cellular proliferation. The AB assay is based on the incorporation of a fluorogenic redox indicator of cell growth in culture. The turnover of AB is a reflection of cell proliferation, and is quantified by measuring the fluorescence in Relative Flourescence Units (RFU). Fluorescence was detected using a fluorescence reader (FLx800 Microplate Fluorescence Reader, BIO-TEK Instruments, Vinooski, Vermont, USA) at 560/20 nm and 620/40 nm at the following time points: immediately after the addition of AB (0 h), then at 3 h, 6 h, 12 h, 24 h, 2 d, 3 d, 4 d and 7 d. Uncultured wells served as a reference. Assays were run in triplicate for each BSM and BSM/EMD composition, and at each time point.

### Statistics

Statistical analysis was performed using the statistical software SigmaStat (Version 3.1.; Systat Software, Inc., Richmond, USA). Means and standard deviations were calculated for each group. Results are shown graphically in a plot (abscissa: point of time, ordinate: RFU values). In order to identify the BSM or BSM/EMD composition showing the greatest proliferation after both 24 h and 7 d, all groups were compared using Bonferroni's t-test. Furthermore, the groups were compared against pure EMD. To verify the differences between BSM without EMD and BSM with EMD, a separate t-test was performed. The outcome each statistical test was considered to be significant with p < 0.05 and highly significant with p < 0.001.

## Results

In general, all of the investigated BSM and BSM/EMD compositions revealed continuous cell proliferation over the observation period, with some significant differences.

After 24 h, the mean values (standard deviation in parentheses) for AB reduction of osteogenic cells cultivated on the various BSM *without *EMD were: control 832 (± 25) RFU, Cerasorb^® ^M 963 (± 16) RFU, Bioresorb^® ^1073 (± 19) RFU, Bio-Oss^® ^863 (± 18) RFU, PerioGlas^® ^705 (± 8) RFU, Straumann^® ^BoneCeramic 963 (± 45) RFU, NanoBone^® ^1088 (± 6) RFU, BONIT^®^matrix 1184 (± 32) RFU.

**Figure 1:**
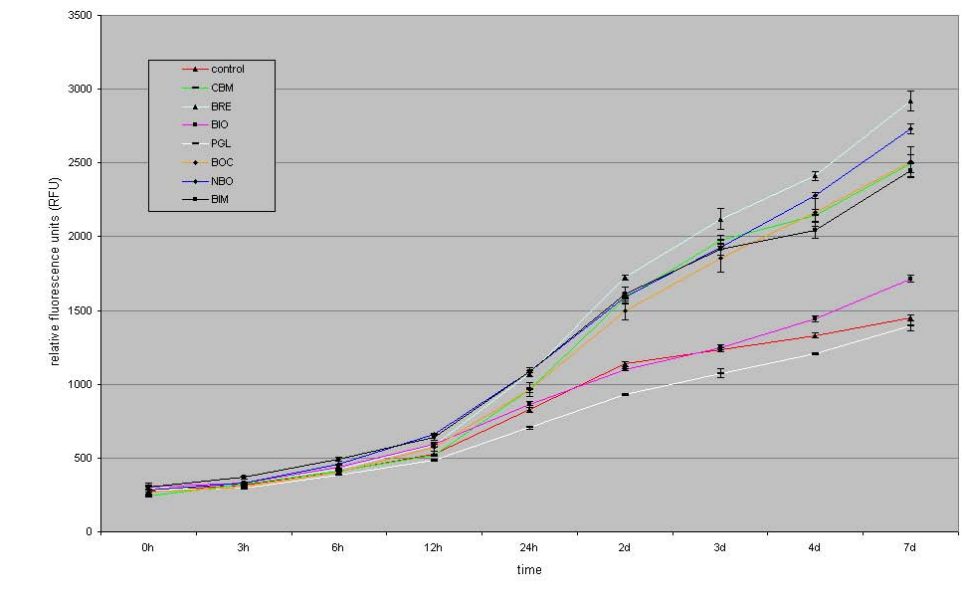
**figure illustrating Bonferroni's t-test for AB reduction of osteogenic cells cultivated on the various BSM compared to the control after 7 d**.

After seven days, the values for AB reduction of osteogenic cells cultivated on the various BSM *without *EMD were: control 1447 (± 20) RFU, BONIT^®^matrix 2450 (± 48) RFU, Straumann^® ^BoneCeramic 2508 (± 100) RFU, PerioGlas^® ^1396 (± 31) RFU, Cerasorb^® ^M 2494 (± 61) RFU, Bioresorb^® ^2921 (± 69) RFU, NanoBone^® ^2733 (± 34) RFU, Bio-Oss^® ^1714 (± 23) RFU. After 7 days, a significant increase in AB reduction, compared to the negative control, was found in decreasing order for Bioresorb^® ^> NanoBone^® ^> Straumann^® ^BoneCeramic > Cerasorb^® ^M > BONIT^®^matrix > Bio-Oss^®^. Furthermore, a slight, but not significant decrease in AB reduction was documented for PerioGlas^® ^(figure 1, table 2).

**Table 2:** Bonferroni's t-test for AB reduction of osteogenic cells cultivated on the various BSM compared to the control after 7 d

Comparison	Diff of Means	t	p
control vs. BRE	1473	26.8	<0.001**

control vs. NBO	1286	23.4	<0.001**

control vs. BOC	1061	19.3	<0.001**

control vs. CBM	1047	19.1	<0.001**

control vs. BIM	1002	18.2	<0.001**

control vs. BIO	267	4.8	0.001*

control vs. PGL	51	0.9	0.936

(BIO = Bio-Oss^®^, NBO = NanoBone^®^, BRE = Bioresorb^®^, CBM = Cerasorb^® ^M, PGL = PerioGlas^®^, BOC = Straumann^® ^BoneCeramic, BIM = BONIT^®^matrix; t = probability; p = p-value; *significant, ** highly significant).

After 24 h, AB reduction values for osteogenic cells cultivated on the various BSM *with *EMD were: control 1055 (± 16) RFU, Cerasorb^® ^M 1034 (± 40) RFU, Bioresorb^® ^1166 (± 13) RFU, Bio-Oss^® ^918 (± 24) RFU, PerioGlas^® ^701 (± 12) RFU, Straumann^® ^BoneCeramic 1045 (± 24) RFU, NanoBone^® ^1181 (± 37) RFU, BONIT^®^matrix 1182 (± 93) RFU.

**Figure 2:**
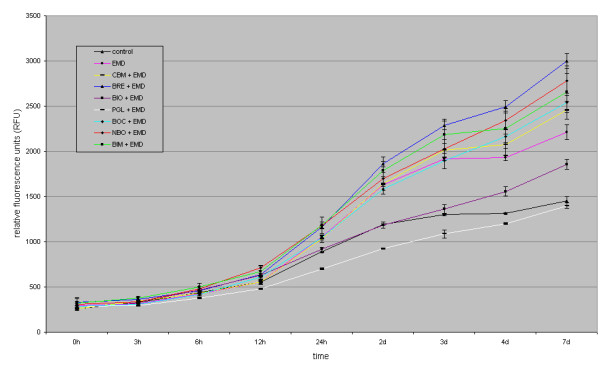
**figure illustrating Bonferroni's t-test for AB reduction of osteogenic cells cultivated on the various BSM compared to the untreated control or EMD after 7 d**.

After 7 days, AB reduction values for osteogenic cells cultivated on the various BSM *with *EMD were: control 1447 (± 80) RFU, EMD 2212 (± 80) RFU, BONIT^®^matrix 2660 (± 206) RFU, Straumann^® ^BoneCeramic 2538 (± 105) RFU, PerioGlas^® ^1399 (± 30) RFU, Cerasorb^® ^M 2456 (± 98) RFU, Bioresorb^® ^2998 (± 83) RFU, NanoBone^® ^2781 (± 162) RFU, Bio-Oss^® ^1854 (± 54) RFU. Compared to the untreated control group, the AB reduction showed a significant increase in descending order for Bioresorb^® ^> NanoBone^® ^> BONIT^®^matrix > Straumann^® ^BoneCeramic > Cerasorb^® ^M > Emdogain^® ^> Bio-Oss^®^. A slight, but not significant decrease in AB reduction was documented for PerioGlas^® ^(figure 2, table 3). Table 3 also provides a comparison between EMD and BSM enriched with EMD.

**Table 3:** Bonferroni's t-test for AB reduction of osteogenic cells cultivated on the various BSM compared to the untreated control or EMD after 7 d

Comparison	Diff of Means	t	p
control vs. BRE + EMD	1544	14.0	<0.001**

control vs. NBO + EMD	1327	12.0	<0.001**

control vs. BIM + EMD	1206	10.9	<0.001**

control vs. BOC + EMD	1084	9.8	<0.001**

control vs. CBM + EMD	1002	9.1	<0.001**

control vs. EMD	758	6.9	<0.001**

control vs. BIO + EMD	400	3.6	0.015*

control vs. PGL + EMD	54	0.4	>1.0

			

EMD vs. PGL + EMD	812	7.0	<0.001**

EMD vs. BRE + EMD	786	6.8	<0.001**

EMD vs. NBO + EMD	569	4.9	0.001*

EMD vs. BIM + EMD	448	3.8	0.009*

EMD vs. BIO + EMD	358	3.1	0.048*

EMD vs. BOC + EMD	326	2.8	0.085

EMD vs. CBM + EMD	244	2.1	0.352

(BIO = Bio-Oss^®^, NBO = NanoBone^®^, BRE = Bioresorb^®^, CBM = Cerasorb^® ^M, PGL = PerioGlas^®^, BOC = Straumann^® ^BoneCeramic, BIM = BONIT^®^matrix; t = probability; p = p-value; *significant, ** highly significant).

**Figure 3:**
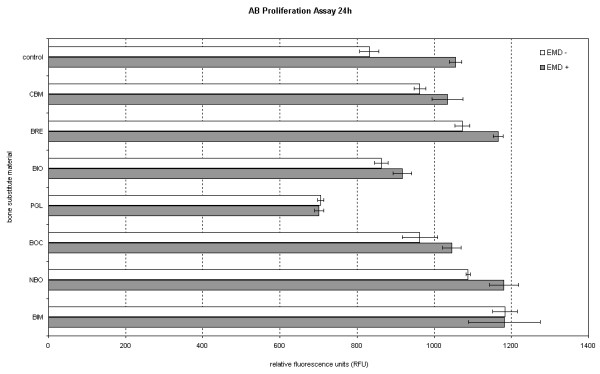
**figure illustrating Comparison of BSM without EMD to BSM + EMD on osteogenic cell proliferation after 24 h and 7 d using the t-test**.

**Figure 4:**
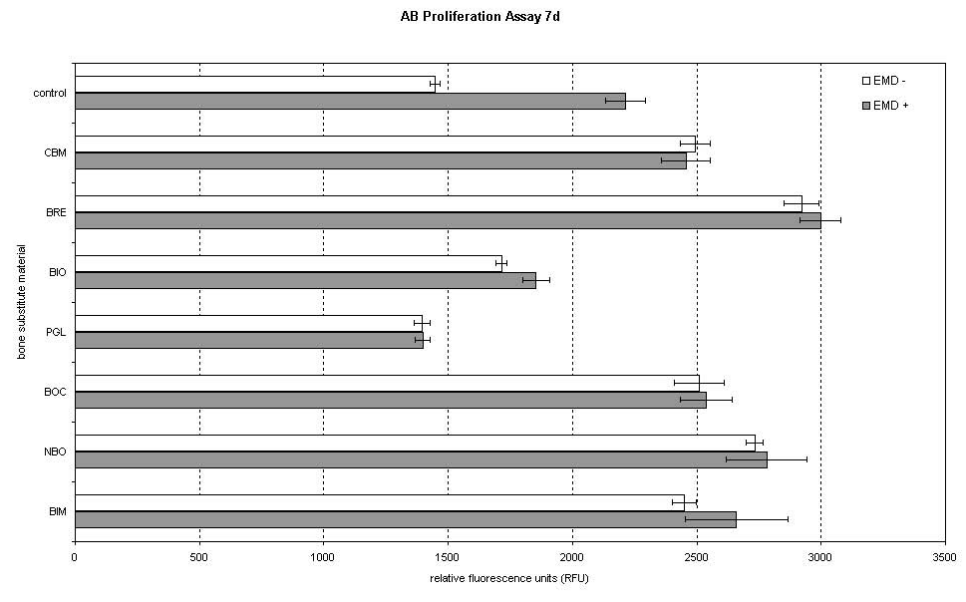
**figure illustrating Comparison of BSM without EMD to BSM + EMD on osteogenic cell proliferation after 24 h and 7 d using the t-test**.

In a comparison of pure BSM and the BSM/EMD composition, all of the BSM, except for PerioGlas^® ^and BONIT^®^matrix, showed an increase in AB reduction values at 24 h with the addition of EMD. For NanoBone^® ^and Bioresorb^®^, the addition of EMD resulted in significantly increased AB reduction values. After 7 days, The only BSM to show a decrease in the AB reduction value with EMD as compared to without EMD was Cerasorb^® ^M. For Bio-Oss^®^, the addition of EMD resulted in a significantly increased AB reduction value (figures 3 and 4, table 4).

**Table 4:** Comparison of BSM without EMD to BSM + EMD on osteogenic cell proliferation after 24 h and 7 d using the t-test

24 h			
**Comparison**	**Diff of Means**	**t**	**p**

BIO vs. BIO + EMD	-54	-2.5	0,06

NBO vs. NBO + EMD	-93	-3.4	0,025 *

BRE vs. BRE + EMD	-93	-5.7	0,004 *

CBM vs. CBM + EMD	-71	-2.3	0,078

PGL vs. PGL + EMD	+4	0.4	0,686

BOC vs. BOC + EMD	-82	-2.2	0,086

BIM vs. BIM + EMD	+2	-1.4	0,231

			

**7d**			

**Comparison**	**Diff of Means**	**T**	**p**

BIO vs. BIO + EMD	-139	-3.3	0.028 *

NBO vs. NBO + EMD	-48	-0.4	0.701

BRE vs. BRE + EMD	-83	-0.3	0.400

CBM vs. CBM + EMD	+38	-0.4	0.663

PGL vs. PGL + EMD	-3	-0.1	0.911

BOC vs. BOC + EMD	-29	-0.2	0.787

BIM vs. BIM + EMD	-210	-1.4	0.231

(BIO = Bio-Oss^®^, NBO = NanoBone^®^, BRE = Bioresorb^®^, CBM = Cerasorb^® ^M, PGL = PerioGlas^®^, BOC = Straumann^® ^BoneCeramic, BIM = BONIT^®^matrix; t = probability; p = p-value; *significant).

## Discussion

When employing alloplastic bone substitute materials (BSM) for guided bone regeneration, the biocompatibility and biological activity of the material used plays an essential role, alongside the distinct physical properties of the graft, like stiffness and stability, for the overall therapeutic success. In this context, the development of an "ideal" synthetic bone graft that fulfils the attributes "biocompatible", "degradable", "osteoconductive", and "osteoinductive" is the focus of recent research. A major issue for the clinical practitioner is whether a bone graft acts as a plain defect filler, or has additional osteoconductive or osteoinductive capacities [29]. The pore size of the BSM plays a crucial role in enhancing the osteoconductive potential of the BSM. Current literature postulates a minimum pore size of between 200-400 μm as necessary for osteoconduction, vascularisation, and formation of mineralised tissue within a scaffold [30-32]. Furthermore, it is known that an increasing number of interconnective pores raises the internal surface area of a BSM, with promotion of the growth of regenerative cells [33].

The assessment of cell proliferation *in vitro *provides valuable clues about substrate biocompatibility. Furthermore, proliferating cells are a precondition for osteoconductivity and osteoinductivity. The BSM investigated in our study represent a cross-section of the currently commercially available grafting materials, reflecting the most popular and well-documented chemical compositions (HA, TCP, bioactive glasses). The sample size of 100 mg of BSM was chosen in order to completely cover the floor of a well in a 24 well plate. This ensured that the majority of the cultivated cells was in close contact with the BSM particles. Our results suggest that none of the grafting materials used in this study has a significantly negative influence on cellular proliferation, as compared to the control. In fact, all but one of the BSM tested led to an increased AB reduction over the observation period of 7d. Only PerioGlas^® ^showed a slight, but not significant decrease in AB reduction, compared to the control. Our findings are, to a certain extent, contrary to former studies [13,34]. Possible explanations might be dissimilarities in the experimental set-up. Furthermore, it should be kept in mind that *in vitro *studies only give a limited reflection of the complex *in vivo *situation.

Although the biomaterial Bio-Oss^® ^showed very good results in various clinical trials [26,35], our *in vitro *investigation showed weaker results for cell proliferation as compared to the other test materials, with the exception of PerioGlas^®^. These findings for Bio-Oss^® ^are in agreement with other *in vitro *studies [13]. In our study, all of the other investigated BSM clearly promoted osteogenic cell proliferation, with the highest values after 24 h for BONIT^®^matrix, and after 7 d for Bioresorb^® ^Macro Pore. Nanocrystalline HA (NanoBone^®^) has been shown to promote other cell lines with osteogenic potential, in a fashion similar to that observed in our study [36].

## Conclusion

In our study, the addition of EMD resulted in an increase in AB reduction for almost all test groups, but significantly for the control, NanoBone^®^, and Bioresorb^® ^Macro Pore after 24 h, as well as for the control, and Bio-Oss^® ^after 7 d. We observed a minimal, EMD-dependent decrease in AB reduction for PerioGlas^® ^after 24 h, and for Cerasorb^® ^M after 7 d. Schwarz et al. observed a benefit in the functionalisation of titanium surfaces with EMD [21]. Altogether, the addition of EMD seems to promote osteogenic cell proliferation to a certain degree. In the routine clinical situation, the benefit of combining BSM and EMD is well established, and scientifically documented [15,16,26].

In our study, we found no clear correlation between the BSM chemical composition or structural properties, and osteogenic cell proliferation - regardless of the addition of EMD. Further research must be conducted to understand the exact modus of interaction between EMD and BSM, e.g. studies of protein release kinetics from BSM with different chemical and structural properties. We could identify promising BSM candidates for enhancing osteogenic cell activity.

## Competing interests

The authors declare that they have no competing interests.

## Authors' contributions

The study design was established by MOK, CR and BA. CR and MOK carried out the *in vitro *experiments and wrote the manuscript. RS performed the data management and data analysis. AK and WG carried out the manuscript editing and manuscript review. All authors read and approved the final version of the manuscript.
